# Emerging salt marshes as a source of *Trichoderma arenarium* sp. nov. and other fungal *bio*effectors for *bio*saline agriculture

**DOI:** 10.1111/jam.14751

**Published:** 2020-07-24

**Authors:** M.‐Y. Ding, W. Chen, X.‐C. Ma, B.‐W. Lv, S.‐Q. Jiang, Y.‐N. Yu, M.J. Rahimi, R.‐W. Gao, Z. Zhao, F. Cai, I.S. Druzhinina

**Affiliations:** ^1^ Fungal Genomics Laboratory (FungiG) Nanjing Agricultural University Nanjing P.R. China; ^2^ Jiangsu Provincial Key Lab of Solid Organic Waste Utilization Jiangsu Collaborative Innovation Center of Solid Organic Wastes Educational Ministry Engineering Center of Resource‐Saving Fertilizers Nanjing Agricultural University Nanjing P.R. China; ^3^ Institute of Chemical, Environmental and Bioscience Engineering (ICEBE) TU Wien Vienna Austria

**Keywords:** Biosaline agriculture, Halotolerant fungi, Plant growth promotion, Rhizosphere, Salt marsh, *Trichoderma arenarium*

## Abstract

**Aims:**

Sustainable agriculture requires effective and safe *bio*fertilizers and *bio*fungicides with low environmental impact. Natural ecosystems that closely resemble the conditions of *bio*saline agriculture may present a reservoir for fungal strains that can be used as novel *bio*effectors.

**Methods and Results:**

We isolated a library of fungi from the rhizosphere of three natural halotolerant plants grown in the emerging tidal salt marshes on the south‐east coast of China. DNA barcoding of 116 isolates based on the rRNA ITS1 and 2 and other markers (*tef1* or *rpb2*) revealed 38 fungal species, including plant pathogenic (41%), saprotrophic (24%) and mycoparasitic (28%) taxa. The mycoparasitic fungi were mainly species from the hypocrealean genus *Trichoderma*, including at least four novel phylotypes. Two of them, representing the taxa *Trichoderma arenarium* sp. nov. (described here) and *T. asperelloides*, showed antagonistic activity against five phytopathogenic fungi, and significant growth promotion on tomato seedlings under the conditions of saline agriculture.

**Conclusions:**

*Trichoderma* spp. of salt marshes play the role of natural biological control in young soil ecosystems with a putatively premature microbiome.

**Significance and Impact of the Study:**

The saline soil microbiome is a rich source of halotolerant *bio*effectors that can be used in *bio*saline agriculture.

## Introduction

Sustainable agriculture requires high yields of crops, which can be achieved if chemical pesticides and synthetic fertilizers are replaced or combined with environmentally friendly *bio*fungicides and *bio*fertilizers (Altomare and Tringovska 2011). In such products, plant‐beneficial micro‐organisms positively influence the microbial community in the rhizosphere and, therefore, protect the plants as biological control agents (BCAs) and stimulate their growth as plant growth‐promoting microbes (PGPMs) (Vessey 2003). Fungi are the essential members of every soil ecosystem, not only as decomposers of organic (mainly plant) matter but also as *bio*trophic associates of plants or other organisms (Trillas and Segarra 2009). Although most fungal–plant interactions are mutualistic (those involving mycorrhizal and endophytic fungi), numerous soil‐borne diseases of plants are also caused by fungi (Redman *et al*. 2001). On the other hand, beneficial interactions between plants and fungi are sensitive to disturbances and require extended period to establish. To date, our understanding of these processes in native soil ecosystems remains incomplete.

Some environmental opportunistic fungi that are capable of efficiently colonizing a variety of substrates can interact with a broad range of organisms without becoming pathogenic to plants or to humans. These fungi can be particularly useful for crop protection (Harman *et al*. 2004). They can rapidly establish in the rhizosphere, compete with plant pathogenic fungi for the resources, and stimulate plant growth (Trillas and Segarra 2009; Harman *et al*. 2019). Several species of the two hypocrealean genera *Clonostachys* (Nygren *et al*. 2018) and *Trichoderma* (Ascomycota, Druzhinina *et al*. 2018) are particularly suitable for such purposes because of their versatile mycoparasitism coupled with plant‐beneficial properties, including production of phytohormone‐like components (Vinale *et al*. 2009; Cai *et al*. 2013) and stimulation of plant systemic resistance (Harman *et al*. 2004; Cai *et al*. 2013). The diversity of these genera is high, but so far, only a handful of species have been used as *bio*effectors in *bio*control formulations (Druzhinina *et al*. 2011; Kubicek *et al*. 2019). However, some of these species also have potentially adverse effects like as mushroom pests (Komoń‐Zelazowska *et al*. 2007; Innocenti *et al*. 2019) or even as pathogens for immunocompromised humans (Sandoval‐Denis *et al*. 2014; Hatvani *et al*. 2019). Therefore, new and safe *bio*effectors are required.

Undisturbed ecosystems can be natural sources of low‐input, multifunctional and renewable microbial *bio*effectors. In nature, when plants germinate from their seed teguments, they associate with the microbes that exist in the surrounding environment. However, only a select subset of this community becomes associated with roots or established in the rhizosphere (Chaparro *et al*. 2014; Santhanam *et al*. 2015). In agriculture, the soil microbial communities are severely disturbed by tilling, culture, weathering and the introduction of various xenobiotics (such as pesticides and fertilizers); thus the soil microbial communities in these ecosystems frequently get reformed (Santhanam *et al*. 2015; Szoboszlay *et al*. 2017; Zhang *et al*. 2017; Hartman *et al*. 2018). For example, a well‐documented agricultural phenomenon is the high frequency of soil‐born disease outbreaks in monocultured crops, which happens due to the unbalanced microbiomes rich in plant pathogenic invertebrates, fungi or bacteria (Santhanam *et al*. 2015; Hartman *et al*. 2018; Wang *et al*. 2019). Some newly formed natural ecosystems may resemble such affected agricultural lands in that they are young and frequently offer similar adverse conditions for microbial communities and plants. Among such ecosystems, the emerging tidal salt marshes in particular may resemble the conditions of *bio*saline agriculture, where saline (sea) water is used for irrigation in arid or coastal areas (Masters *et al*. 2007; Ayyam *et al*. 2019). Native plants in these conditions may be prone to diseases because of the extremely limited vegetation diversity (equivalent to monoculture), the disturbance from seawater intrusion, and the salinization of the soil surface (Li *et al*. 2018; Ayyam *et al*. 2019). Interestingly, in most of such seemingly simple natural ecosystems, even single pioneer species of plants stay healthy (Li *et al*. 2018; Ayyam *et al*. 2019).

Hence, we hypothesize that the wild plants growing in emerging tidal salt marshes may have queried the soil microbial community to assist them, namely they may have recruited some native *bio*effectors as root associations in response to challenges, such as biotic (pests) and abiotic (salinity, oligotrophy and climate) challenges. In this study, we investigate the possibility of beneficial interactions between wild plants and their associated fungi in an emerging tidal salt marsh screening for native *bio*effectors potentially suitable for agricultural use.

## Materials and Methods

### The study area and sample collection

**Figure 1:**
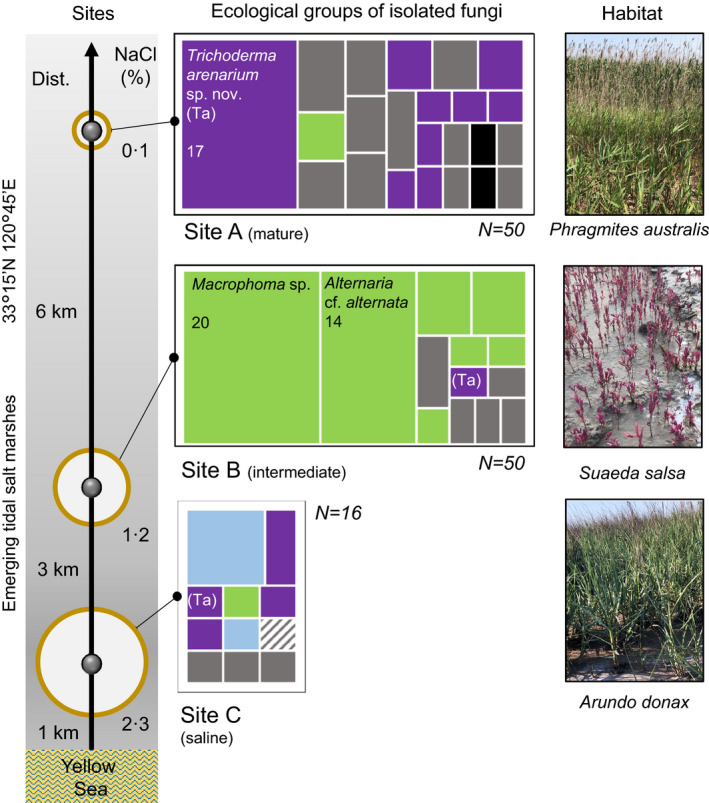
Ecological diversity of rhizospheric fungi in emerging salt marshes. The white cycles show the salinity of the sampled sites (Table 1). The tree plots show the number of fungal strains belonging to the major ecological groups isolated from every site. The dominant taxa are named; the number corresponds to the number of isolates. Habitats are shown using pictures taken on the day of the sampling (Image credit: F. Cai). (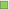) Herbivore, incli. endophyte; (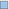) Aquatic, incl. marine; (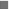) Saprotroph; (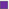) Fungivore; (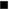) Carnivore. [Colour figure can be viewed at wileyonlinelibrary.com]

The coastal tidal flat (33°15′N, 120°45′E) in the Jiangsu province of China, spread over 6·53 × 10^5^ ha, represents the largest tidal wetland in eastern Asia (Long *et al*. 2016; Li *et al*. 2018). The costal mud flat in Dafeng Nature Reserve is the central part of this area, which keeps growing by 50‐200 m per year towards the Yellow Sea. The area is under the influence of the northern subtropical monsoon climate, with a mean annual temperature of 15°C and a mean annual rainfall of 1058 mm (Long *et al*. 2016; Li *et al*. 2018; Jiang *et al*. 2019). Halophytic vegetation like *Arundo donax* (Poaceae) and *Suaeda salsa* (Chenopodiaceae) are the pioneer plants on this saline soil, followed by the common reed *Phragmites australis* (Poaceae) mixed with cogongrass *Imperata cylindrica* (Poaceae), which are the dominant species after the salinity drops (Li *et al*. 2018). Therefore, for our study, we selected three plants from three sites to sample their rhizosphere soil: *P. australis* (site A), *S. salsa* (site B) and *A. donax* (site C). The sampling sites are shown in Fig. 1. Nine rhizosphere soil samples located 200 m apart were collected for each plant in June 2019, as described by Cai *et al*. (2015). Briefly, the whole plant was carefully removed from the soil, and the bulk of the soil was removed from the roots by shaking the plant vigorously. The soil still adhering on the roots was considered as the rhizosphere soil. The rhizosphere soil samples were then stored separately in sterilized bags and transported to the laboratory on ice. Soil chemical properties, including organic matter (OM) content and available phosphorus (AP), were measured as described in our previous study (Jiang *et al*. 2019). Soil pH and electrical conductivity (EC) were measured in a 1 : 5 (w/v) suspension at 25°C. Soil nitrate nitrogen (NN) and ammoniacal nitrogen (AN) content was analysed with a continuous‐flow analyser (AutoAnalyzer 3, Bran + Luebbe GmbH, Germany) as described previously (Cai *et al*. 2015; Jiang *et al*. 2019).

### Estimation of bacterial and fungal abundance and isolation of fungi

The standard 10‐fold dilution plating method was adopted for screening and isolation of bacteria and fungi from the collected soil samples. Specifically, 5 g of each soil sample was suspended in 45 ml of sterilized distilled water and was serially diluted for another 1000 folds. From the last two dilutions, 100 *μ*l of the soil suspension was spread over the surface of LB plates (Thermo Fisher Scientific) for bacteria and PDA (BD Difco, Becton, Dickinson and Company, Franklin Lakes, NJ, USA) supplemented with 400 *μ*g ml^−1^ chloramphenicol plates for fungi. The colony‐forming units (CFU) on each plate were recorded separately for bacteria and fungi. Distinct fungal colonies were purified with the single‐cell separation method (Zeb *et al*. 2019).

### DNA barcoding and phylogenetic analysis

All isolated fungi were DNA barcoded using the primary (ITS1 and 2 rRNA; White *et al*. 1990), and the secondary (the RNA polymerase II subunit B gene, *rpb2*; Liu *et al*. 1999 and the translation elongation factor 1‐alpha, *tef1*; Samuels *et al*. 2002) markers were applied when needed. For this purpose, fungal genomic DNA was extracted using a Phire Plant Direct PCR kit (Thermo Scientific) according to the manufacturer’s instructions. All the obtained isolates were sequenced for the ITS1 and 2 rRNA using the ITS1 and ITS4 primers (White *et al*. 1990). The *Trichoderma* strains were further sequenced for the *rpb2* and *tef1* with the primer pairs of fRPB2‐5f and fRPB2‐7cr (Liu *et al*. 1999) and EF1 and EF2 (O’Donnell *et al*. 1998) respectively.

All sequences were aligned with MUSCLE that integrated in the mega 5 software for each locus separately and were grouped to phylotypes (Tamura *et al*. 2011). Unique phylotypes were subjected to the sequence similarity search tool blastn against the NCBI GenBank database (http://www.ncbi.nlm.nih.gov, February 2020). A species was assigned to the query strain when sequences of ITS1 and 2 rRNA were found to be identical to the type or published reference strains. Strains with the possibility of being putatively new species and ambiguous cases were assigned at the genus level. Fungi identified as *Trichoderma* by means of ITS1 and 2 rRNA were then further investigated by the analysis of the diagnostic fragment of *tef1* and of *rpb2* using a sequence similarity search against the NCBI GenBank and *Tricho*BLAST (http://www.isth.info; Kopchinskiy *et al*. 2005) databases. The closely related sequences found in the GenBank database were retrieved.

For phylogenetic analysis, all the obtained sequences were aligned using muscle 3.8.31 integrated in AliView 1.23 (Larsson 2014). Isolates from the same soil sample sharing identical sequences of the three DNA barcode markers were treated as one fungal haplotype (genet). The sequence similarity search using NCBI blastn with the ITS1 and 2 and the *rpb2* and *tef1* sequences was performed to retrieve the vouchered sequences of the closely related strains and the identified species in the public database. The corresponding sequences of the type or published reference strains of the most closely related species were also downloaded based on the best BLAST hits. Alignment files were then generated for each marker, and the flanking areas were manually trimmed. The Bayesian information criterion was used to select the best fit model with ModelFinder (Kalyaanamoorthy *et al*. 2017) implemented in IQ‐TREE 1.6.12 (Nguyen *et al*. 2015). Maximum likelihood (ML) analysis was computed with IQ‐TREE. ML bootstrap proportions were computed for 1000 replicates. The obtained phylogenetic trees were viewed in FigTree v1.4.4 and edited in Corel Draw 2018.

### Phenotypic assays

For the assessment of macro‐morphology, fungi were inoculated on three different media—PDA, SNA (synthetic low nutrient agar, Nirenberg 1976) and CMD (4% cornmeal + 2% dextrose; Jaklitsch 2009)—and incubated at 25°C with 12 h of illumination and 12 h of darkness for 7 days. The macro‐morphology of the strains was recorded with a Canon EOS 70D (equipped with a Canon 100 μm macro lens) under white light. The micro‐morphology was investigated using a Leica DMi8 microscope (Leica, Wetzlar, Germany) and a cryo‐scanning electron microscope (cryo‐SEM, Quorum PP3010T integrated onto a Hitachi SU8010 FE‐SEM, Japan). In the cryo‐SEM, the fungal culture was rapidly frozen in liquid nitrogen slush, fractured at −140°C and coated with 5 nm of platinum.

Salinity and pH tolerance assays for fungi were performed in (Costar^TM^96‐well microplates, Corning, NY, USA). Two microlitres of spore suspension (10^8^ spores ml^−1^) of each strain were inoculated into 198 *μ*l of 30% Murashige Skoog basal salt mixture medium (MS, Sigma‐Aldrich, USA) supplemented with 1% glucose (MSG), and incubated at 25°C in darkness. The salinity of the MSG medium was previously adjusted with NaCl to concentrations at 0, 0·5, 1·0 and 1·5 mol l^−1^. In another assay, the pH gradient was set up as pH values at 5·0, 7·0, 8·0 and 9·0. Growth was monitored as O.D.750 nm of each well every 12 or 24 h using a Spectra Max iD3 microplate reader (Molecular Devices, USA).

### Fungal dual confrontation assays

The antagonistic activity of the selected *Trichoderma* isolates was investigated by dual confrontation assays, as described in Zhang *et al*. (2019), against the following fungi. From Ascomycota: *Alternaria* cf. *alternata* TUCIM 10217 (Pleosporales), *Macrophoma* sp. TUCIM 10254 (Botryosphaeriales), *Pestalotiopsis fici* TUCIM 5788 (Xylariales) (Druzhinina *et al*. 2018), *Fusarium odoratissimum* TUCIM 4848 (Hypocreales) (named as *Fusarium oxysporum* f.sp. *cubense* 4, Foc4 in Zhang *et al*. 2019), and from Basidiomycota: *Rhizoctonia solani* TUCIM 3753 (Cantharellales) (Derntl *et al*. 2017). *Alternaria* cf. *alternata* TUCIM 10217 and *Macrophoma* sp. TUCIM 10254 were isolated in this study (see below). Briefly, a plug of fresh culture (6 mm) of an opponent fungus was placed 1 cm from the edge of the PDA plate (9 cm diameter) and incubated at 25°C in darkness for 24 h. Then a similar culture plug of the *Trichoderma* sp. was placed on the opposite edge of the same plate. The fungi were allowed to grow under the above incubating condition for 14 days, and the fungal combat on each plate was recorded with a Canon EOS 70D camera.

### Plant growth promotion experiment

To analyse the growth promotion effect of the selected *Trichoderma* spp. on plant, a pot experiment was carried out with tomato seedlings (*Solanum lycopersicum* L. cv. HEZUO903). Three seedlings, all 3 weeks old, were planted in each pot containing 300 g of a mixture (w/w = 1 : 1) of vermiculite (1–3 mm) and perlite (1–3 mm) at a pH of 6·0. The salinity of the growth substrate was adjusted by adding NaCl to 0·5% and 0·75%, representing medium and high salinity stress conditions, respectively, and using a 0% NaCl group as the control. Three millilitres of *Trichoderma* spore suspension (10^8^ spores ml^−1^) were inoculated to the roots in each pot. Ten millilitres of 10% MS irrigation was applied every 2 weeks. The plants were allowed to grow at 25°C under cycled illumination conditions (light : darkness = 16 : 8) for 6 weeks. At the end of the experiment, data regarding plant growth and health, including plant height, fresh and dry biomass, and the SPAD value for measuring the leaf chlorophyll content, were recoded for each seedling (*N* = 12 per each treatment). Root development was measured using a root scanner (Epson Perfection v700 Photo, Seiko Epson, Japan), as described previously (Cai *et al*. 2013).

### Statistical analysis

The means and the standard deviations of the data were calculated using PASW 18.0 (IBM Corporation, Chicago, IL, USA). Multiple comparisons were performed using the analysis of variance (anova) and Duncan’s multiple range test (*P* = 0·05) integrated in PASW 18.0. The heatmap was plotted in r v3·2.2.

## Results

### Study area and sampling sites

The study area, Dafeng Nature Reserve, is located at the east costal region of China, which faces the Yellow Sea. The area consists of the emerging salt marshes (Solonchak, IUSS Working Group WRB, FAO 2015) that formed 50 years ago and is still growing towards the sea due to the large amount of sediment carried by the Yellow River and the Yangtze River (Li *et al*. 2018). The reserve is a typical coastal mud flat, characterized by a gentle slope formed with successive saline soil. The land offers a unique opportunity to study hydromorphic soil development, vegetation succession and microbiome assembly (Long *et al*. 2016). The natural vegetation succession in this area starts with the giant cane *A. donax* (Poaceae) close to the sea shore, followed by the highly halotolerant native red plant *S. salsa* (Chenopodiaceae), and the cosmopolitan fire‐adapted grass *I. cylindrica* (Poaceae). In the relatively mature ecosystems several kilometres inland, the marshes are colonized by the common reed *P. australis* (Poaceae). Large colonies of *P. australis*, *S. salsa* and *A. donax*, occupying several square kilometres, undisturbed by human activities, were selected as sampling sites A, B and C respectively (Fig. 1). The comparative analysis of soil properties revealed high pH (ca. 8·5) at all three sites, and no difference in ammoniacal nitrogen (AN) or available phosphate (AP) between the three sampling sites (*P *> 0·05, Table 1). The nitrate nitrogen (NN) and organic matter (OM) slightly increased with increased distance from the sea (*P* < 0·05), but remained comparable. However, the soils in the three sites had very different salinization and electrical conductivity (EC, an indicator of the total salinity of soil) values, with the lowest salinity at site A and the highest at site C (Fig. 1 and Table 1).

**Table 1:** Soil chemical properties of the sampling site

Sample site	pH value		Electrical conductivity		Organic matter		Ammoniacal nitrogen		Nitrate nitrogen		Available phosphate	
			(dS m^−1^)		(g kg^‐1^)		(mg kg^‐1^)					
	Mean	SD	Mean	SD	Mean	SD	Mean	SD	Mean	SD	Mean	SD
Site A	8·51^a^	0·41	3·37^a^	1·31	**5·77** ^a^	1·27	4·08^a^	1·91	**7·53** ^a^	1·53	8·11^a^	1·88	
Site B	8·39^a^	0·28	23·35^b^	2·26	**4·56** ^a^	1·07	4·38^a^	2·69	3·19^b^	0·86	9·54^a^	1·2	
Site C	8·48^a^	0·24	**37·98** ^c^	8·7	2·99^b^	1·28	2·64^a^	1·04	2.5^b^	0·24	10·4^a^	3·08	

Statistically significantly different values are labelled with different letters (*N* = 9, ANOVA, *P* < 0·05). The bold font highlights the statistically significantly largest values among the sites.

### Fungal abundance correlates with soil properties

**Figure 2:**
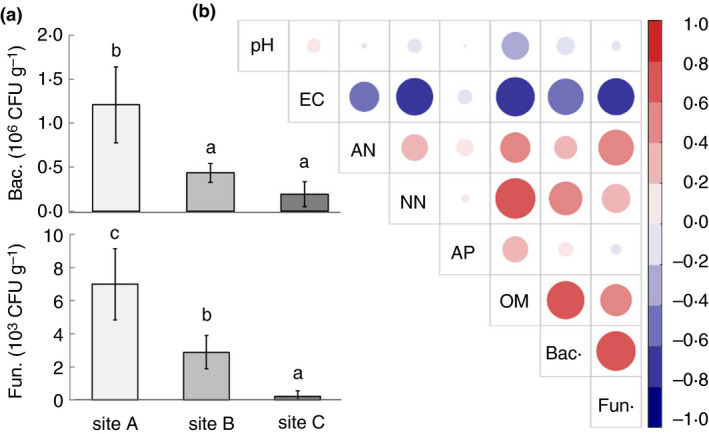
Microbial abundance (a) and its correlations (b) with the chemical properties of the sampled soil. Bac.: bacterial amount; Fun.: fungal amount; EC: soil electrical conductivity; AN: ammoniacal nitrogen; NN: nitrate nitrogen; AP: available phosphate; OM: organic matter. The bars shown in (a) followed by the same letter are not statistically significantly different (anova, *P *> 0·05). The correlation coefficient is represented by the size of the circle. [Colour figure can be viewed at wileyonlinelibrary.com]

The abundance of cultured bacteria and fungi decreased significantly from site A to site C (Fig. 2a). The most closely related soil properties to microbial abundance were EC, OM and NN; on the other hand, pH values, AN and AP were not clearly related to it (Fig. 2b). Specifically, both bacterial and fungal abundances were positively correlated with OM and NN, and were negatively correlated with soil EC values.

We isolated 50 fungal strains from the rhizosphere of *P. australis* (site A) and 50 from the rhizosphere of *S. salsa* (site B), and only 16 from the rhizosphere of *A. donax* (site C) (Fig. 1, Table 2). DNA barcoding based on the internal transcribed spacers (ITS1 and 2) of the ribosomal RNA gene cluster revealed in total 38 fungal phylotypes. Of these, 65 isolates could be reliably identified by the sequence similarity to the vouchered isolates deposited in public databases and confirmed by taxonomic literatures (Table 2), and 20 more isolates were identified after sequencing additional DNA barcoding markers, such as fragments of *tef1* and *rpb2* genes (Table 2). In total, 85 isolates were thus identified by species, but the taxonomic position of 31 additional isolates (like four *Trichoderma* spp., *Coniothyrium* sp. TUCIM 1024, a new hypocrealean strain TUCIM 10250, and others, Table 2) remained undefined, suggesting the existence of putatively new taxa or species that have no corresponding DNA barcodes in public databases.

**Table 2:** The fungal species from coastal saline soils identified in this study and their identification based on the reference strains

N	Species	Ecol. group	Sites			DNA Barcoding of the ref. strain			Identification			
			A	B	C	TUCIM	Marker	GenBank accession	Ref. strain	GenBank accession	Similarity (%)	Coverage (%)
Pleosporales, Dothideomycetes												
1	*Alternaria* cf. *alternata*	Herb	–	14	–	10217	ITS 1 and 2	MT217111	XJ‐KT131‐2	MK616289	100	100
2	*A*. cf. *chlamydospora*	Herb	–	3	–	10231	ITS 1 and 2	MT217112	**CBS 491.72**	NR_136039	100	100
3	*Phoma* cf. *betae*	Herb	–	1	–	10291	ITS 1 and 2	MT217113	CBS 111.85	KY940781	99	100
4	*Westerdykella dispersa*	sapr	–	–	1	10332	ITS 1 and 2	MT217114	**CBS 297.56**	NR_111187	100	100
5	*Stemphylium* cf. *lycopersici*	Herb	–	1	1	10299	ITS 1 and 2	MT217115	**CBS 122639**	NR_155002	100	100
6	*Phaeosphaeria spartinae*	aquat	–	–	5	10286	ITS 1 and 2	MT217116	CBS 254.64	AF439506	99	98
7	*Pyrenochaetopsis tabarestanensis*	sapr	2	–	–	10294	ITS 1 and 2	MT217117	IBRC M 30051	NR_155636	100	99
8	*Coniothyrium* sp.	myc	1	–	–	10243	ITS 1 and 2	MT217118	NRRL 66000	KM056318	100	100
9	*Paraconiothyrium estuarinum*	myc	1	–	–	10279	ITS 1 and 2	MT217119	**CBS 109850**	NR_137669	100	96
10	*Macrophoma* sp.	Herb	–	20	–	10254	ITS 1 and 2	MT217120	TXc‐4	HQ262514	100	100
11	*Cladosporium* cf. *silenes*	sapr	2	2	–	10239	ITS 1 and 2	MT217121	**CPC 14253**	NR_119855	100	100
Hypocreales, Sordariomycetes												
12	*Trichoderma* sp.	myc	1	–	2	10329	*tef1*	MT242300				
13	*Trichoderma* sp.	myc	1	–	1	10325	*tef1*	MT242301				
14	*T. caerulescens*	myc	2	–	–	10321	ITS 1 and 2	MT217122	CBS 130011	NR_134432	100	99
15	*Trichoderma* sp.	myc	2	–	–	10328	*tef1*	MT262967				
16	*T. arenarium* sp. nov.	myc	17	1	1	10301 10302	ITS 1 and 2	MT217123				
17							*rpb2*	MT242310				
18							*tef1*	MT242303				
19	*T. asperelloides*	myc	1	–	–	10320	*rpb2*	MT242313	**GJS 04‐111**	GU198281		
20							*tef1*	MT242304		GU198294		
21	*Trichoderma* sp.	myc	1	–	1	10323	*tef1*	MT242305				
22	*Fusarium* cf. *falciforme*	Herb	–	1	–	10247	ITS 1 and 2	MT217124	**CBS 475.67**	NR_164424	100	100
23	*F*. cf. *proliferatum*	Herb	2	–	–	10248	ITS 1 and 2	MT217125	ZmH10	MG228402	100	100
24	*F*. cf. *equiseti*	Herb	–	3	–	10244	ITS 1 and 2	MT217126	DYL6Z	MN589985	100	100
25	*Paracremonium binnewijzendii*	aquat	–	–	1	10280	ITS 1 and 2	MT217127	**CBS 143277**	NR_157491	99	100
26	*Lecanicillium saksenae*	carn	1	–	–	10251	ITS 1 and 2	MT217128	**IMI 179841**	NR_111102	98	100
27	*Acremonium strictum*	sapr	–	–	1	10296	ITS 1 and 2	MT217129	**CBS 346·70**	NR_111145	100	100
28	*Sarocladium terricola*	sapr	1	–	–	10297	ITS 1 and 2	MT217130	CBS 134.71	HG965038	100	100
29	*Parasarocladium* sp.	–	–	–	1	10250	ITS 1 and 2	MT217131	**CBS 142.62**	NR_161112	95	100
30	*Purpureocillium* sp.	sapr	2	–	–	10292	ITS 1 and 2	MT217132	**CBS 284.36**	NR_111432	99	91
31	*Scopulariopsis* cf. *cordiae*	carn	1	–	–	10298	ITS 1 and 2	MT217133	**CBS 138129**	NR_132958	98	100
Eurotiales, Eurotiomycetes												
32	*Penicillium* cf. *oxalicum*	sapr	–	1	–	10282	ITS 1 and 2	MT217134	**NRRL787**	NR_121232	100	100
33	*P*. cf. *citrinum*	sapr	–	1	–	10281	ITS 1 and 2	MT217135	**NRRL1841**	NR_121224	100	100
34	*P*. cf. *steckii*	sapr	3	–	–	10283	ITS 1 and 2	MT217136	CBS 130380	MH865790	100	100
35	*Aspergillus* cf. *fumigatus*	sapr	1	–	1	10234	ITS 1 and 2	MT217137	**ATCC 1022**	NR_121481	100	100
36	*A*. cf. *niger*	sapr	2	–	–	10236	ITS 1 and 2	MT217138	**ZmH27**	MG228419	100	100
37	*A*. cf. *templicola*	sapr	1	–	–	10238	ITS 1 and 2	MT217139	CBS 138181	NR_135456	99	100
Mucoromycotina												
38	*Mucor* cf. *hiemalis*	sapr	2	–	–	10277	ITS 1 and 2	MT217140	**CBS 201.65**	NR_152948	99	99
39	*M*. cf. *racemosus*	sapr	–	1	–	10276	ITS 1 and 2	MT217141	GZ20190123	MN726736	100	100
40	*Mortierella alpina*	sapr	1	1	–	10272	ITS 1 and 2	MT217142	ATCC 16266	GU319989	100	100
41	*M*. cf. *amoeboidea*	sapr	2	–	–	10274	ITS 1 and 2	MT217143	CBS 889.72	NR_111579	96	98

Herb: herbivore; sapr: saprotroph; aquat: aquatic; myc: mycoparasitism; carn: carnivore. Type reference strains are shown in bold.

Although all the plants sampled appeared healthy, the fungi isolated from rhizosphere of *S. salsa* (site B) were predominantly species that are known to be plant pathogenic (*Macrophoma* sp., *Alternaria* spp., *F. equiseti*, and others; Table 2). Fungi isolated from the two other sites, site A and site C, were ecologically equally versatile, although the habitats differed in salinity. Thus, the rhizosphere of *P. australis* (site A) was dominated by a putatively new phylotype of *Trichoderma*, *T*. sp. TUCIM 10301, followed by four other putatively new *Trichoderma* spp., *T. asperelloide* *s* and *T. caerulescens*, but also the two other mycoparasitic fungi (*Coniothyrium* sp. TUCIM 10243 and *Paraconiothyrium* *estuarinum* TUCIM 10279), and a variety of common saprotrophic fungi, such as species of *Aspergillus*, *Penicillium* (Eurotiales), and some common Mucoromycotina (*Mucor* spp., *Mortierella* spp.; Table 2, Fig. 1). Similarly, a mixture of mycoparasitic and saprotrophic fungi was recovered from the samples of site C. As this site is located near the costal line, we also found a few aquatic or marine fungi there (*Phaeosphaeria spartinae* from Pleosporales and hypocrealean *Paracremonium binnewijzendii*). Interestingly, the diversity recovered from the invasive environmentally opportunistic plant species, the common reed and the giant cane, was rich in the environmental opportunistic species of fungi, that are, *Trichoderma* spp., *Aspergillus* spp. and *Mucor* spp.

### Two *Trichoderma* strains tolerate high salinity and alkaline pH

**Figure 3:**
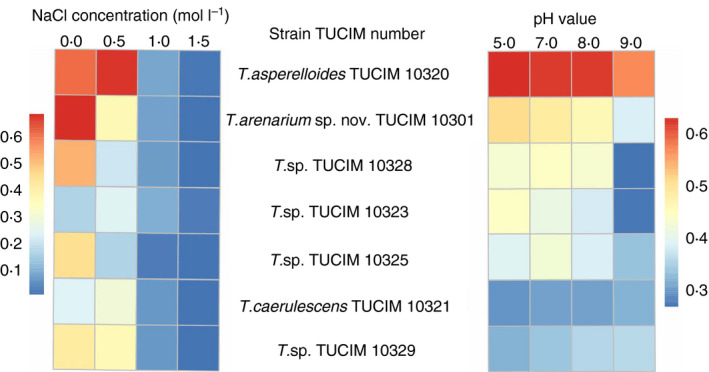
Heat map showing the fungal growth (O.D.750 nm) cultured in 30% Murashige Skoog basal salt mixture medium (including 1% glucose, MSG) amended with different concentrations of NaCl (left) or adjusted to different pH values (right). Numbers appeared near the colour intensity bar refer to the growth of the fungus that was measured at O.D. 750 nm [Colour figure can be viewed at wileyonlinelibrary.com]

*Trichoderma* spp. are well‐recognized plant‐beneficial fungi that are used as *bio*effectors in *bio*fungicides for controlling fungal diseases in crops (biocontrol) and/or in *bio*fertilizers for plant growth promotion (see review in Druzhinina *et al*. 2011). The diversity of the isolated *Trichoderma* strains in this study consisted of seven phylotypes (Table 2), of which two could be reliably identified to the species level (*T. asperelloides* and *T. caerulescens*; see below) and five were putatively new taxa. Therefore, in order to select possible *bio*effective strains that can be used under the conditions of *bio*saline agriculture, we first tested the tolerance of the strains to high salinity and alkaline pH, the parameters that represent or extend the conditions of their native habitat. One strain per each of the seven phylotypes was randomly selected for these tests. Based on the results given in Table 1 (that the salinity of the three sites ranged from 0·36 to 2·3%, with pH consistently around 8·4–8·5), four gradients of each stress factor were set (Fig. 3).

As shown in Fig. 3, strain TUCIM 10320 grew significantly more in the presence of 0·5 mol l^−1^ NaCl (2·9% NaCl, close to the natural salinity of site C) and 1·0 mol l^−1^ NaCl, compared to the other strains and to itself when grown under nonsaline conditions (anova, *P* < 0·05). Therefore, we assume that this strain is halophilic, while the others are halotolerant. Several strains were sensitive to NaCl (Fig. 3). Furthermore, the growth of strains *T*. sp. TUCIM 10301 and *T*. sp. TUCIM 10329 was significantly greater than the growth of other strains *T*. sp. TUCIM 10328, *T*. sp. TUCIM 10323, *T*. sp. 10325 and *T. caerulescens* TUCIM 10321 under the condition of 0·5 mol l^−1^ NaCl. However, the growth of all the strains tested declined dramatically when the NaCl concentration reached 1·5 mol l^−1^ (ca. 8%).

The halophilic strain *T. asperelloides* TUCIM 10320 best adapted to alkaline pH values, followed by strains *T*. sp. TUCIM 10301 and *T*. sp. TUCIM 10328. The other *Trichoderma* spp. strains, TUCIM 10323, TUCIM 10325, TUCIM 10329 and *T. caerulescens* TUCIM 10321, showed comparatively weaker growth than the above three strains under the test conditions. Based on their adaptability to the two stress factors tested, strains *T. asperelloides* TUCIM 10320 and *T*. sp. TUCIM 10301 were selected for subsequent experiments.

### Phylogenetic and phenotypic analysis reveals a new *Trichoderma* species

**Figure 4:**
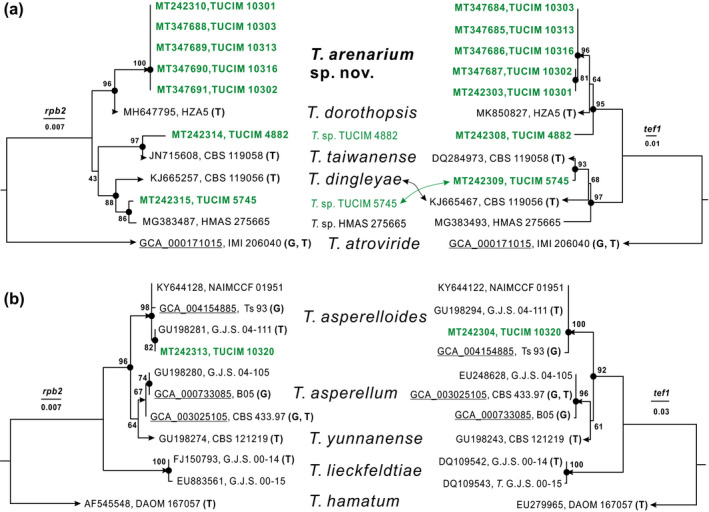
Phylogenetic identification of strains TUCIM 10301 (a) and TUCIM 10320 (b) via the loci of *rpb2* and *tef1* and maximum likelihood analysis. The green font highlights the strains first used in this study. The black circles indicate the nodes supported by IQ‐TREE ultrafast bootstrap >70. Arrows indicate branches that lead to species. GenBank accessions of each locus per strain are given, followed by the strain name. T: type strain; G: genome‐sequenced strain (genome accession numbers are underlined). [Colour figure can be viewed at wileyonlinelibrary.com]

To reveal the taxonomic position of the *bio*effective *T*. sp. TUCIM 10301, which by far dominated our culture library from site A, we first performed the sequence similarity search using the blastn tool. The results showed that TUCIM 10301 had identical ITS1 and 2 phylotype to strains TRI2 (GenBank: KF691740) and BBA 65450 (GenBank: KF691740), both deposited as *Trichoderma viridescens* isolated from mulberry in China. No identical *rpb2* or *tef1* (the fourth large intron to the fifth intron) records were found in the NCBI database. However, *T. viridescens* cannot be identified by means of the ITS1 and 2 DNA barcode (Jaklitsch *et al*. 2013). The most similar sequences were from the strain HZA5 of a recently described species *T. dorothopsis* (deposited as *Trichoderma* sp. AA‐2019, Tomah *et al*. 2020), which was also isolated from soil in China, and which shared a 98.77% *rpb2* (GenBank: MH647795) and a 97·52% *tef1* (GenBank: MK850827) phylotype with TUCIM 10301 (*E*‐value was equal to zero for both comparisons). The similarity of strain TUCIM 10301 to the most closely related defined species *Trichoderma dingleyae* and *Trichoderma taiwanense* was, respectively, 97·29 and 97·12% for *rpb2*, and 85·53 and 91·06% for *tef1*. This indicates that TUCIM 10301 belongs to the *Trichoderma* Section of this genus. The taxonomy report obtained from this search revealed that besides *T. dorothopsis*, *T. dingleyae* and *T. taiwanense*, the query strain was also related to *T*. sp. strain IQ 11 (namely TUCIM 4882 from South America) and *T*. sp. TUCIM 5745 from South‐east Asia. The ML phylogram (Fig. 4a) constructed with *rpb2* sequences demonstrated that the five isolates, formed a statistically supported clade separate from the most closely related genetic neighbours (*T. dorothopsis* *, T. dingleyae*, *T. taiwanense*, *T*. sp. TUCIM 5745 and *T*. sp. TUCIM 4882). Similar tree topology supporting the presence of this clade was also obtained for the *tef1* phylogenetic marker (Fig. 4a). Thus, the isolates represented by *T*. sp. TUCIM 10301 met the criteria of the genealogical concordance phylogenetic species recognition concept (Taylor *et al*. 2000), as they form distinct clades on the phylograms constructed based on the two unlinked loci (*rpb2* and *tef1*) and also have a unique ITS1 and 2 rRNA phylotype. Therefore, we recognize it as a new species described below as *T. arenarium* sp. nov.

Strain TUCIM 10320 was found to be identical to the type strain of *T. asperelloides* G.J.S. 04‐111 (Samuels *et al*. 2010) when the *rpb2* and *tef1* loci were used, as shown in Fig. 4b, and thus it was identified as *T. asperelloides*.

### *Trichoderma arenarium* sp. nov. and *T. asperelloides* combat a variety of plant pathogenic fungi

**Figure 5:**
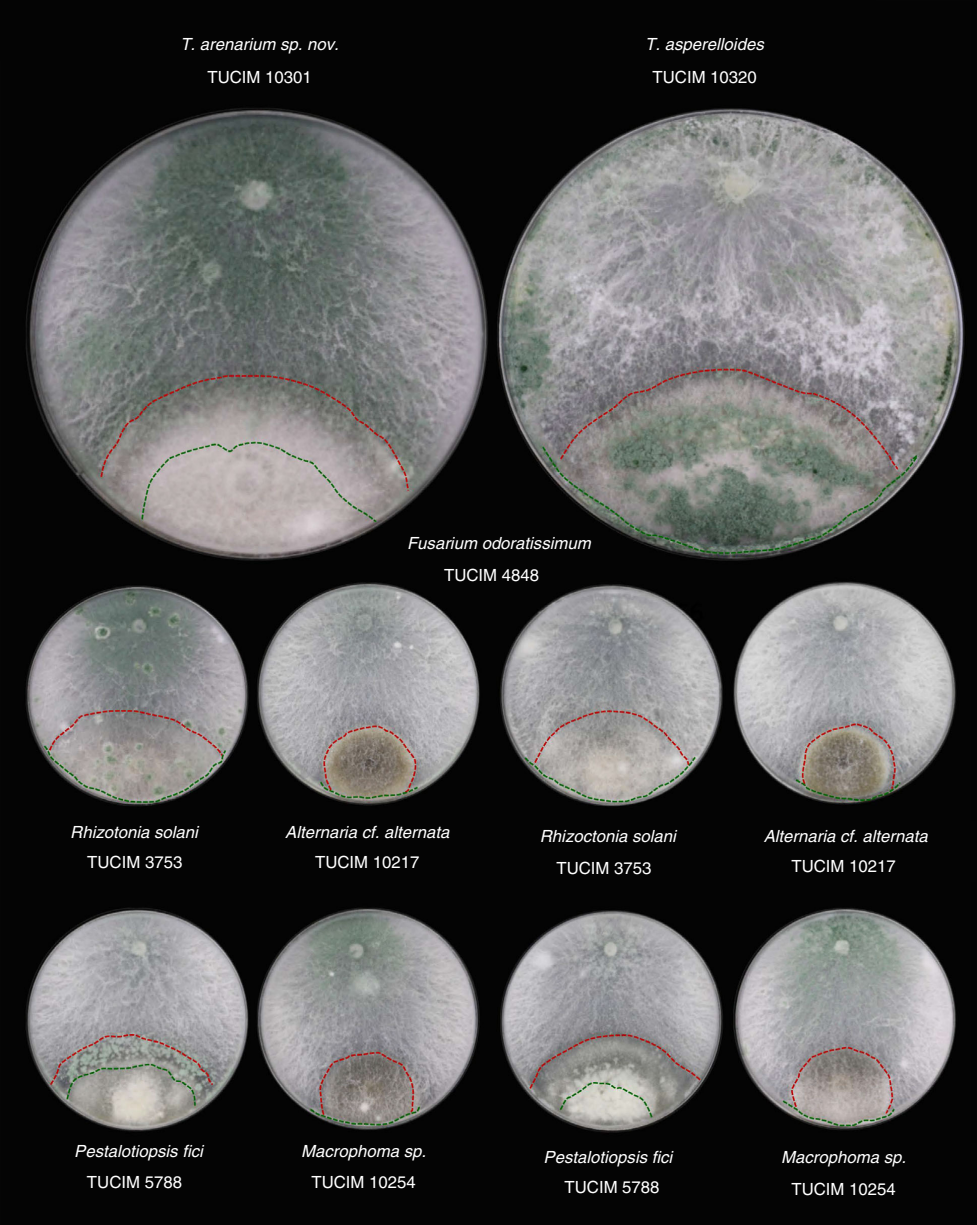
Dual confrontation assays between the selected *Trichoderma* strains and five plant pathogenic fungi. Fungi were cultivated on PDA plates, and images were recorded after 14 days of incubation at 25°C in darkness. The green dashed line shows the colony edge of *Trichoderma*. The red dashed line shows the colony edge of the partner fungus. The plate diameter is 9 cm. [Colour figure can be viewed at wileyonlinelibrary.com]

In order to investigate whether the isolated *Trichoderma* strains have potential in biocontrol of plant pathogens, dual confrontation assays were done between the two *Trichoderma* spp. (TUCIM 10301 and 10320) and five phytopathogenic fungi. We used two fungi isolated in this study (*Alternaria* cf. *alternata* TUCIM 10217 and *Macrophoma* sp. TUCIM 10254) and three other reported pathogenic fungi, *F. odoratissimum* TUCIM 4848, *R. solani* TUCIM 3753 and *Pestalotiopsis fici* TUCIM 5788. The results showed that *T. arenarium* sp. nov. TUCIM 10301 and *T. asperelloides* TUCIM 10320 efficiently combated and overgrew the two sympatric fungi as well as *R. solani* TUCIM 3753 (Fig. 5). However, these two *Trichoderma* strains both showed weaker antagonism against *P. fici* TUCIM 5788. *T. asperelloides* TUCIM 10320 could not combat *P. fici* and remained in a ‘deadlock’ stage (where the growth of one fungus is limited by another; see more about fungal ‘deadlock’ in Zhang *et al*. 2019). *T. arenarium* sp. nov. TUCIM 10301 formed a clear conidia ring surrounding the *P. fici* colony. As for *F. odoratissimum* TUCIM 4848, *T. arenarium* sp. nov. TUCIM 10301 overgrew on it partially, while *T. asperelloides* TUCIM 10320 completely combatted this fungus and formed dense conidia above it. This response is relatively rare for *Trichoderma* spp. (Zhang *et al*. 2019).

### *Trichoderma arenarium* sp. nov. and *T. asperelloides* promote plant growth in conditions of high salinity

**Figure 6:**
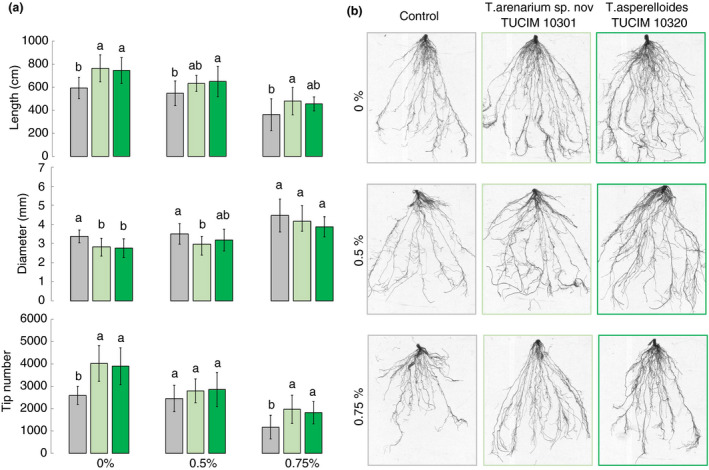
The effect of the two bioactive *Trichoderma* strains on tomato roots cultivated under saline conditions. The bars shown in (a) followed by the same letter are not statistically significantly different (anova, *P *> 0·05). (b) Representative root scanning images of tomato seedlings from each treatment. Grey bars: seedlings without *Trichoderma* inoculation, the control; Light green bars: seedlings inoculated *T. arenarium* sp. nov. TUCIM 10301; Dark green bars: seedlings inoculated with *T. asperelloides* TUCIM 10320. [Colour figure can be viewed at wileyonlinelibrary.com]

To test whether the obtained *T. arenarium* sp. nov. and *T. asperelloides* strains can be used for plant growth promotion in *bio*saline agriculture, a pot experiment was carried out with a model plant (tomato, *S. lycopersicum* L.) under three different salinity conditions (0, 0·5 and 0·75% NaCl). The evaluation of tomato seedlings (Table 3) showed that the inoculation of *T. arenarium* sp. nov. TUCIM 10301 and *T. asperelloides* TUCIM 10320 significantly (anova *, P* < 0·05) promoted the biomass and the height of the seedlings compared to the control at both medium (0·5%) and high (0·75%) salinity conditions, as well as at the nonsalinity condition. Specifically, the *Trichoderma* inoculations increased the dry weight of the seedlings by 30–81% under the salty conditions and by 41–107% under the nonsalinity conditions relative to the *Trichoderma‐*free control. Moreover, the effect of the *Trichoderma* inoculations on SPAD reads (which measure the relative chlorophyll content in leaves) suggested that *Trichoderma* played a role in eliminating the chlorophyll reduction that normally caused by high salinity. As salinity has a severe negative effect on roots (Ayyam *et al*. 2019), we also used a root scanner to evaluate root development in a detailed way. The results (Fig. 6) showed that the *Trichoderma* inoculations significantly (anova, *P* < 0·05) promoted the total root length and the number of root tips compared to the control, while correspondingly, the root diameters were smaller in the *Trichoderma* treatments than in the control. The difference between the two *Trichoderma* strains on plant growth was not significant under saline conditions (anova, *P*> 0·05), but under zero‐salinity conditions, *T. asperelloides* TUCIM 10320 showed significantly (anova, *P* < 0·05) stronger promotion than *T. arenarium* sp. nov. TUCIM 10301 on plant growth.

**Table 3:** Plant growth of tomato seedlings with and without *Trichoderma* inoculation under different salinity conditions

NaCl (%)	Strain	SPAD		Plant height (cm)		Fresh weight (g per plant)		Dry weight (g per plant)	
		Mean	SD	Mean	SD	mean	SD	Mean	SD
0	Control	36·49^b^	1·76	35·29^b^	3·21	5·02^c^	0·72	0·29^c^	0·05
	*T. arenarium* sp. nov. TUCIM 10301	**38·88** ^a^	1·12	**39·51** ^a^	4·7	6·88^b^	1·21	0·41^b^	0·07
	*T. asperelloides* TUCIM 10320	**38.6** ^a^	2·18	**41·55** ^a^	2·64	**9·44** ^a^	1·08	**0.6** ^a^	0·1
0·5	Control	34·64^c^	2·85	25·18^b^	2·49	4·26^b^	1·12	0.3^b^	0·08
	*T. arenarium* sp. nov. TUCIM 10301	**48.3** ^a^	3·94	**29·07** ^a^	2·03	**5·29** ^a^	0·63	**0·41** ^a^	0·08
	*T. asperelloides* TUCIM 10320	43·47^b^	3·6	25·96^b^	2·82	**4·82** ^ab^	0·98	**0·39** ^a^	0·09
0·75	Control	34·69^b^	3·65	21·43^a^	2·11	3·29^b^	1·05	0·21^b^	0·09
	*T. arenarium* sp. nov. TUCIM 10301	**43·48** ^a^	4·89	23·11^a^	2·41	**4.4** ^a^	1·07	**0·38** ^a^	0·12
	*T. asperelloides* TUCIM 10320	**43·11** ^a^	7·03	**22·73** ^a^	1·98	**4·37** ^a^	0·54	**0·34** ^a^	0·07

Statistically significantly different values are labelled with different letters (*N* = 12, ANOVA, *P* < 0·05). The bold font highlights the statistically significantly largest values among the treatments.

### Taxonomy

**Figure 7:**
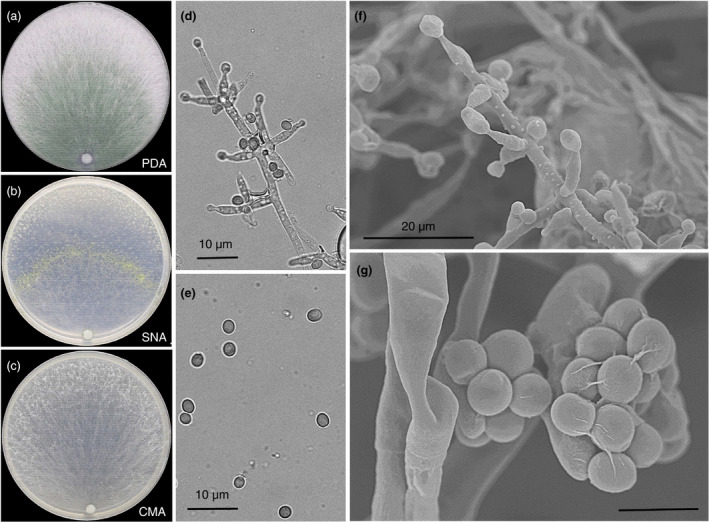
The morphology of *Trichoderma arenarium* sp. nov. The colonies grown on PDA, SNA and CMA media are shown in panels (a), (b) and (c) respectively. The fungus was incubated at 25°C with 12 h light and 12 h darkness for 7 days. (d) Branching phialides and conidiophores, (e) Conidia, (f) cryo‐SEM of phialides and conidiophores, (g) cryo‐SEM of hyphae and conidia clumps. [Colour figure can be viewed at wileyonlinelibrary.com]

***Trichoderma arenarium*** F. Cai, M.Y. Ding &I. S. Druzhinina, sp. nov. (Fig. 7).

Syn: *Trichoderma arenerea*

MycoBank No.: (MB 835845)

Teleomorph: None known

Colony radius on PDA after 48 h at 25°C in intermittent light: 80–85 mm. Mycelia bundled and white. No growth at 35°C. Conidia forming within 96 h on PDA at 25°C. Colonies grown on PDA at 25°C for 1 week under a cycled photoperiod (light:darkness = 12 h:12 h) filled the Petri plate with a continuous lawn of conidia that were abundant and associated with 1–2 mm diam. pustules. On SNA, the conidia formed in concentric rings. On CMD, the mycelium was loose; no conidia found. No diffusing pigment or distinct odour was noticed. Conidiophores comprised a distinct central axis, 3·0–3·5 μm wide, finely warted from which secondary branches arose, mostly unilateral, consisting of one or two cells on the tip; all branches terminating in a single phialide. Phialides were flask shaped, more or less swollen in the middle. Conidia were subglobose to ellipsoidal; most were dark green, 1·8–2·5 μm diam (*n* = 30).

Holotype: China, isolated from rhizosphere soil of *P. australis* grown in coastal saline land, Dafeng, Jiangsu province, June 2019, M.Y. Ding, TUCIM 10301, CGMCC 19611.

Additional culture examined: isolated from rhizosphere soil of *P. australis* and *S. salsa* grown in coastal saline land, Dafeng, Jiangsu province, June 2019, M.Y. Ding, TUCIM 10302, TUCIM 10303, TUCIM 10313 and TUCIM 10316.

Etymology: ‘arenarium’ refers to the sandy and muddy salt marsh ecosystem where the fungus was detected. However, ‘arenerea’ was used for a patent application (Patent accession number: 202010377558.7, China).

## Discussion

Soil arguably harbours the world’s most diverse microbiome (Jansson and Hofmockel 2020). Plants anchor in the soil by their roots and recruit particular microbial taxa from the soil marketplace as potential partners (Turner *et al*. 2013; Santhanam *et al*. 2015). Our understanding on this process and the factors governing behind is very limited for most microbial taxa. As for fungi, besides the interactions of plants with mycorrhizal and phytopathogenic fungi (which have been frequently studied), the mechanisms driving the nonpathogenic fungi in rhizosphere remain unknown (Redman *et al*. 2001; Harman *et al*. 2019). In this study, by screening the cultured fungi in the rhizospheres of several pioneer plant species found in the emerging tidal salt marshes, we inadvertently recapitulated a common biological question: why do some *Trichoderma* species preferentially enrich in rhizosphere, or even colonize on roots? Similarly to what frequently happens in agriculture (Trillas and Segarra 2009; Szoboszlay *et al*. 2017; Hartman *et al*. 2018), the perennation of some Poaceae species colonizing the tidal salt marshes results in an accumulation of some specific phytopathogenic fungi (e.g. *Macrophoma* sp., *Alternaria* spp., and *Fusarium* spp.) in their rhizosphere. Consequently, *Trichoderma* spp., as a mycoparasite (Kubicek *et al*. 2011; Druzhinina *et al*. 2018), may trace fungi, including phytopathogenic ones in such ecosystems, thus becoming root associated. Although we are not able to exclude other possible factors attracting *Trichoderma* spp. to roots, it could be evidence of *bio*control happening in nature. Throughout evolutionary history, native wild plants growing in this ecosystem may have been querying their soil microbial community to assist them in dealing with potential challenges (like the phytopathogen accumulation here). And this may help us empower crops to perform the same by screening the native *bio*effectors for the specific plants or for the established ecosystem.

The results of this study confirm the initial hypotheses and show that some of the isolated strains can be used as *bio*effectors in agriculture, since the *Trichoderma* spp. found in the sample area significantly promoted plant growth under various salinity conditions and were able to antagonize the sympatric and allopatric plant pathogenic fungi. Besides, five of the seven *Trichoderma* phylotypes found could be putatively recognized as new species, suggesting that there may be a huge potential source of new microbial taxa hidden in these young extreme ecosystems. Similar observations were noted in several other studies of marine *Trichoderma* (Gal‐Hemed *et al*. 2011; Vacondio *et al*. 2015), which also detected putatively new phylotypes.

The new species *T. arenarium* sp. nov., which is described here having the closest sibling *T. dorothopsis* (type strain HZA5, not isolated in this study but also found in the soil of the Yangtze River basin, Tomah *et al*. 2020), may be a local species associated with the coastal soils in this region, as no other strain records were found in other locations so far. However, as the present sampling land is formed from the large amount of sediment carried by the Yellow River and the Yangtze River, the *Trichoderma* strains may have also been introduced from upstream habitats, like the Gobi Desert or the Loess Plateau, where the massive sediments of the Yellow Sea originate.

Saline soils are widespread all over the world, accounting for 7–8% of the Earth’s surface (Artiola *et al*. 2019). Coastal saline soil, such as that found in salt marshes, represents a subclass of saline soils, and is recognized as an important potential land resource for agricultural development (Long *et al*. 2016; Ayyam *et al*. 2019). However, crop growth in such areas is usually very limited, due to the high salinity and low nutrient availability in the soil (Ayyam *et al.,* 2019). Regardless of breeding salt‐tolerant plant cultivars, in this study, we showed that a possible alternative is to identify *bio*effectors from local or similar ecosystems for use in saline soil agriculture. The work of Hingole and Pathak (2016) also highlighted the saline soil microbiome as a rich source of halotolerant *bio*effectors. In our case, the *S. salsa* rhizosphere was found to be unsuitable as a source of novel *bio*effective strains, as it maintained a very different mycoflora. Compared to the isolates from the *P. australis* and *A. donax* samples, the screening of the *S. salsa* rhizosphere yielded mainly phytopathogenic fungi, suggesting the possibility of plant‐specific selection in fungal enrichment. Moreover, among the fungivorous fungal genera, *Trichoderma* is the largest taxon, with many ubiquitously distributed species (Kubicek *et al*. 2011; Druzhinina *et al*. 2018; Kubicek *et al*. 2019). Most species (80%) (Druzhinina *et al*. 2011; Friedl and Druzhinina 2012; Kubicek *et al*. 2019) have been found to be relatively rare, but a few dozens of species are known to be present in soils all over the world and are considered environmental opportunists with cosmopolitan distribution. In the present work, the most frequent *Trichoderma* species was *T. arenarium* sp. nov., followed by several other species within the section *Trichoderma*, rather than the *T. harzianum* sensu lato group that frequently found in soil (Druzhinina *et al*. 2010; Chaverri *et al*. 2015), indicating that *T. arenarium* sp. nov. is well adapted to the local niche. Therefore, the study also demonstrates that native *bio*effectors may be more effective than the allopatric strains in developing local biocontrol products. As for coastal saline lands, *bio*saline agriculture offers a solution to the imbalance between the limited arable land and the growing human population by using salt‐affected soil and water (Ayyam *et al*. 2019). This requires the selection of suitable halophytes not only for the plants to be grown but also for the possible associated microorganisms 2020.

## Author contributions

FC and ISD conceived and designed the study. MD, XM, BL, SJ, YY, MJR, RG, ZZ, FC and ISD carried out the experiments. FC, MD and ISD carried out the data analysis and prepared the figures. FC, WC and ISD wrote and revised the manuscript. All authors read and approved the manuscript.

## Conflict of interest

The authors declare no competing interests.
